# Enzymatic Versus Energy-Based Degradation of the Cross-Linked Hyaluronic Acid Macromolecule: A Comparative Physicochemical Study of Hyaluronidase, Focused Ultrasound and Laser Irradiation

**DOI:** 10.3390/polym18172077

**Published:** 2026-08-27

**Authors:** Anna Deda, Sławomir Wilczyński, Selin Sagbas Suner, Aneta Ostróżka-Cieślik, Anna Stolecka-Warzecha, Nurettin Sahiner

**Affiliations:** 1 Department of Practical Cosmetology and Skin Diagnostics, Faculty of Pharmaceutical Sciences in Sosnowiec, Medical University of Silesia, 40-084 Katowice, Poland; adeda@sum.edu.pl; 2 Department of Basic Biomedical Science, Faculty of Pharmaceutical Sciences in Sosnowiec, Medical University of Silesia, Jedności Street 8b, 41-208 Sosnowiec, Poland; astolecka@sum.edu.pl; 3 Department of Chemistry, Faculty of Sciences, Canakkale Onsekiz Mart University, Terzioglu Campus, Canakkale 17100, Turkey; sagbasselin@gmail.com; 4 Department of Pharmaceutical Technology, Faculty of Pharmaceutical Sciences in Sosnowiec, Medical University of Silesia, Jedności Street 8b, 41-208 Sosnowiec, Poland; 5 Department of Chemical Engineering, Faculty of Engineering, Canakkale Onsekiz Mart University, Canakkale 17100, Turkey; nsahiner@fgcu.edu; 6 Department of Bioengineering, U. A. Whitaker College of Engineering, Florida Gulf Coast University, Fort Myers, FL 33965, USA

**Keywords:** hyaluronic acid, cross-linked hydrogel, macromolecular degradation, molecular weight, hyaluronidase, Nd:YAG laser, photothermal scission

## Abstract

Hyaluronic acid (HA) is a high-molecular-weight glycosaminoglycan whose cross-linked hydrogels are widely used as injectable fillers; their controlled degradation is clinically important but, in practice, achievable only enzymatically with hyaluronidase. Here we compared the enzymatic, ultrasonic and photothermal degradation of a single cross-linked HA macromolecular network and characterised the resulting structural, thermal and chromatographic changes. A cross-linked HA hydrogel (Regenyal Idea, 25 mg/mL) was embedded in an ex vivo porcine skin matrix and treated in six groups: untreated control; hyaluronidase; microfocused ultrasound (7 MHz, 3 mm focal depth); and irradiation with 1064 nm Nd:YAG, diode or alexandrite lasers. Degradation was characterised by FT-IR spectroscopy, thermogravimetric analysis (TGA) and high-performance liquid chromatography (HPLC). FT-IR showed retention of the HA backbone with partial loss of the cross-linked network, while TGA revealed treatment-dependent decreases in thermal stability. By HPLC, hyaluronidase released the most soluble HA (58.2 ± 2.9% at day 1; ~88% plateau by day 2). Among the energy-based methods, only the 1064 nm Nd:YAG laser produced comparable degradation (57.3 ± 5.2%), whereas microfocused ultrasound, the diode and the alexandrite lasers released little soluble HA (3.2–5.3%). The wavelength dependence is consistent with a water-mediated photothermal scission of the HA chains. These findings identify long-pulsed 1064 nm irradiation as an effective non-enzymatic route to degrade the cross-linked hyaluronic acid macromolecule, with hyaluronidase remaining the reference standard.

## 1. Introduction

Over the past decade, injectable fillers based on cross-linked hyaluronic acid (HA) have become one of the most frequently performed procedures in aesthetic medicine. According to the international survey of the International Society of Aesthetic Plastic Surgery (ISAPS), the number of HA-based filler procedures performed worldwide rose from 4,312,037 in 2022 to 5,564,866 in 2023, placing them among the leading non-surgical interventions [1]. The popularity of HA reflects its biocompatibility, the reversibility of its effect, and a broad spectrum of indications ranging from the correction of wrinkles and restoration of tissue volume to the reshaping of facial contours. Paradoxically, the very ease with which these treatments are now offered and performed translates into a growing number of patients presenting with complications or dissatisfaction with the aesthetic result, in whom removal of the injected material becomes necessary.

Removing filler from the tissues, however, proves considerably more difficult than is commonly assumed. Contrary to the assumption of complete biodegradation within several months, magnetic resonance imaging (MRI) studies document the presence of cross-linked HA in the tissues for many years after injection; in a review of 33 MRI studies the filler remained detectable in every patient for at least two years, and in individual cases for as long as 15 years [2]. Clinical reports confirm its long-term persistence and its tendency to migrate [3]. Prolonged retention of the material favours late complications such as filler displacement, foreign-body granulomas, delayed-onset hypersensitivity reactions, and biofilm-associated infections [3]. The durability of cross-linked, compartmentalised deposits, together with the limited enzymatic accessibility of large boluses, means that spontaneous elimination is often incomplete and therapeutic intervention is difficult [2].

At present, the only widely used method for actively dissolving HA fillers is enzymatic degradation with hyaluronidase [4,5]. By catalysing the hydrolysis of the glycosidic bonds of the HA chain, this enzyme enables both the correction of non-acute complications—the Tyndall effect, nodules, asymmetries, and excess material—and the emergency treatment of vascular complications, including vascular occlusion and the threat of vision loss [4]. Hyaluronidase is therefore regarded as the mainstay, and effectively the standard of care, for removing hyaluronic acid: in practice the great majority of HA fillers are removed enzymatically, and no equally established alternative currently exists [4,5]. Nevertheless, despite its firmly established position, hyaluronidase has important limitations that have prompted an active search for new methods.

The first limitation is a lack of selectivity. The enzyme does not distinguish between injected and endogenous material, so that an excessive dose also degrades the skin’s native HA, leading to collapse and uneven loss of volume, particularly in regions of thin skin or limited native HA reserves [5,6]. The second concerns dosing and predictability: controlled data on dosage remain limited, and the enzyme requirement varies with the product and the degree of cross-linking—highly cross-linked and monophasic fillers respond more slowly and demand higher doses, repeated injections, and adjunctive techniques such as massage [4,6,7]. The third, and clinically the most serious, is immunogenicity. As a heterologous protein, most often of animal origin, hyaluronidase carries a risk of hypersensitivity reactions ranging from local erythema and pruritus to rare immunoglobulin-E-mediated anaphylaxis; patients sensitised to hymenoptera venom, in which hyaluronidase is an allergenic component, are especially vulnerable, and pre-treatment skin testing, although recommended, is rarely performed in practice [6,8,9]. Together, the non-selectivity, the variable and product-dependent dosing, and the risk of allergic reactions justify the search for alternative, non-enzymatic methods of degrading HA fillers.

A particularly promising direction is the use of ultrasound energy. Hyaluronic acid undergoes depolymerisation under ultrasound through a mechanism that combines the mechanical forces of acoustic cavitation with the action of free radicals (OH· and H·) generated during the collapse of cavitation bubbles; these radicals cleave the glycosidic bonds between glucuronic acid and N-acetylglucosamine units, shortening the chains, and the process is more efficient the higher the initial molecular weight of the polymer [10]. Independently of this sonochemical degradation, high-intensity focused ultrasound (HIFU)—the modality employed in devices marketed for skin tightening such as Ulthera—produces a rapid rise in temperature at the focal point, of the order of 56–90 °C, exceeding the threshold for thermal destabilisation of HA and likewise leading to degradation of the filler [11]. To date this phenomenon has been described mainly as an undesirable interaction threatening filler integrity when energy-based devices and HA are used concurrently [11,12], yet the same property may be deliberately harnessed as a tool for the controlled removal of the material.

The second energy-based approach investigated in the present work is laser irradiation. Lasers act on HA principally through a photothermal mechanism: energy absorbed by tissue water and by the filler deposit is converted into heat, and once the temperature exceeds the threshold of thermal stability the cross-linked HA network is destabilised and the polymer chains are broken [11]. Because water is the dominant chromophore, the efficiency of this effect depends strongly on wavelength and on the depth at which the energy is deposited, which motivates the comparison of lasers operating in different regions of the spectrum. In this study three clinically established laser types were applied to hyaluronic acid: the neodymium-doped yttrium–aluminium–garnet (Nd:YAG) laser, the diode laser, and the alexandrite laser. These wavelengths differ markedly in their absorption by water and melanin and in their tissue penetration, so that each may interact with an HA deposit in a distinct way. Earlier experimental work on the interaction of common lasers with HA fillers—for example in a porcine model—was designed to demonstrate the safety of laser treatment in the vicinity of filler, and reported variable or minimal interaction depending on the device and settings [13]; the targeted use of a laser to modify or reduce a filler deposit, as in the intralesional Nd:YAG treatment of filler granulomas, has been described only in isolated reports [14]. The question of whether, and under what conditions, these lasers can serve as a controlled, non-enzymatic means of degrading HA therefore remains open. Taken together, the available literature has examined ultrasound– and laser–filler interactions largely from a safety standpoint or in isolated case reports, and no previous study has directly compared enzymatic degradation with several energy-based modalities applied to the same cross-linked filler under a common analytical protocol [10,11,12,13,14]; the present work was designed to address this gap.

The aim of the present work is to evaluate and compare the ability of different physical and enzymatic methods to degrade injectable hyaluronic acid. Alongside enzymatic degradation with hyaluronidase, taken as the reference method, the study assesses two energy-based approaches: irradiation with high-intensity focused ultrasound (Ulthera) and irradiation with three types of laser—Nd:YAG, diode, and alexandrite. The objective is to determine whether these energy-based modalities can constitute an effective, non-enzymatic alternative to hyaluronidase for the removal of hyaluronic acid fillers, thereby broadening the therapeutic options available in the management of filler-related complications.

## 2. Materials and Methods

### 2.1. Study Design

This was a controlled, comparative ex vivo study designed to evaluate and compare the effects of enzymatic, ultrasonic, and laser treatments on the structural integrity and degradation behaviour of injectable cross-linked hyaluronic acid (HA). A single, commercially available cross-linked HA filler was injected into freshly excised porcine skin and allocated to six treatment groups: (1) untreated HA (control); (2) HA exposed to hyaluronidase; (3) HA sonicated with microfocused ultrasound; and (4–6) HA irradiated with a neodymium-doped yttrium–aluminium–garnet (Nd:YAG) laser, a diode laser, and an alexandrite laser, respectively. After treatment, all samples were retrieved and subjected to an identical analytical protocol comprising Fourier-transform infrared (FT-IR) spectroscopy, thermogravimetric analysis (TGA), and high-performance liquid chromatography (HPLC), in order to characterise the chemical, thermal, and quantitative degradation of the polymer under each condition.

### 2.2. Materials

The test material was Regenyal Idea (Regenyal Laboratories S.r.l., San Benedetto del Tronto Italy; distributed in Poland by Fenice Sp. z o.o., Warsaw, Poland), hereafter referred to as HA-standard. Regenyal Idea is a single-phase, cross-linked hyaluronic acid dermal filler with a total HA concentration of 25 mg/mL, of which approximately 8% is present as free, non-cross-linked intercalated HA. According to the manufacturer, the product is obtained through a patented single-phase cross-linking process that combines hyaluronic acid chains of differing molecular weights to form a network of high structural complexity that effectively resists degradation; the filler is extensively purified to remove residual cross-linking agent, which limits post-injection inflammatory reactions, erythema and oedema. It is supplied in a 1 mL prefilled syringe, and its labelled indications include the restoration of facial volume (zygomatic and sub-zygomatic regions, nasolabial folds, oral commissures and chin) and the correction of superficial, moderate and deep wrinkles. A single production lot was used throughout the study to avoid batch-to-batch variability. The enzymatic agent was hyaluronidase, reconstituted in phosphate-buffered saline (PBS) immediately before use. Phosphate-buffered saline (pH 7.4), sulfuric acid, and all other reagents were of analytical grade. Dialysis membranes were used for the degradation assays.

### 2.3. Ex Vivo Tissue Model and Sample Preparation

An ex vivo porcine ear model was used as a skin surrogate, given its close histological and biophysical resemblance to human facial skin. A total of six freshly excised porcine ears were obtained from a local abattoir from animals slaughtered for the food chain, for which no separate ethical approval was required; the ears were transported to the laboratory at 4 °C and used within 24 h of slaughter, being kept at 4 °C until processing. Before use, the ears were rinsed with 0.9% (*w*/*v*) saline, the hair was trimmed, the skin was disinfected, and the tissue was allowed to equilibrate to room temperature for approximately 30 min. Using a 27-gauge needle, 0.3 mL of HA-idea was injected into each ear at the junction between the reticular dermis and the subcutaneous tissue, corresponding to a depth of approximately 2–3 mm below the skin surface, so as to reproduce the anatomical plane in which such fillers are clinically deposited. Each treatment condition was applied to three replicate depots (biological triplicate), giving a total of eighteen depots; the depots were spaced at least 1.5 cm apart and distributed across the ears to minimise position-related variability. After injection, the boli were left to equilibrate in situ for 30 min before the corresponding treatment was applied.

### 2.4. Treatment Groups

The six samples were treated as follows.

**Sample 1: Control (untreated HA).** The HA-idea depot received no further intervention and served as the reference for all analytical comparisons.

**Sample 2: Enzymatic treatment (hyaluronidase).** The HA-idea depot was infiltrated with hyaluronidase solution. For the quantitative degradation assay, 0.035 g of hyaluronidase was dissolved in 2 mL of PBS (bovine testicular hyaluronidase, Hylase^®^ “Dessau”, 150 IU per vial; Riemser Pharma GmbH, Greifswald, Germany), and 1 mL of HA-idea was mixed with 2 mL of the enzyme solution; the corresponding enzyme-free control consisted of 1 mL of HA-idea suspended in 2 mL of PBS.

**Sample 3: Microfocused ultrasound (Ulthera).** The HA-idea depot was sonicated using a microfocused ultrasound device (Ulthera) equipped with a 7 MHz transducer with a focal depth of 3 mm, so that the focal zone coincided with the plane of the HA depot. The system used was the Ulthera System (Ulthera Inc./Merz Aesthetics, Mesa, AZ, USA) fitted with a DS 7-3.0 DeepSEE transducer (7 MHz, 3.0 mm focal depth). A total of 22 lines were delivered over the HA depot at an energy of 0.45 J per line, giving a cumulative delivered energy of approximately 9.9 J; each line was applied as a single discrete exposure, and the treatment was completed in approximately 60 s.

**Sample 4: Nd:YAG laser (1064 nm).** The HA-idea depot was irradiated with a neodymium-doped yttrium–aluminium–garnet laser operating at a wavelength of 1064 nm, with a pulse duration of 150 ms; 10 consecutive pulses were delivered. The Nd:YAG laser was applied at a fluence of 120 J/cm^2^ through a 7 mm spot at a repetition rate of approximately 1 Hz; dynamic cryogen-spray cooling of the epidermis was active, while the long 150 ms pulse and the deep penetration of the 1064 nm wavelength allowed the sub-dermal depot to heat despite surface cooling.

**Sample 5: Diode laser (808 nm).** The HA-idea depot was irradiated with a diode laser at a fluence of 25 J/cm^2^ and a pulse duration of 25 ms; 10 consecutive pulses were delivered. The diode laser emitted at 808 nm; a 12 mm spot was used at a repetition rate of approximately 1 Hz, with the handpiece’s integrated sapphire contact cooling (tip temperature ≈ 5 °C) active to protect the epidermis.

**Sample 6: Alexandrite laser (755 nm).** The HA-idea depot was irradiated with an alexandrite laser at a fluence of 25 J/cm^2^ and a pulse duration of 20 ms; 10 consecutive pulses were delivered. The alexandrite laser emitted at 755 nm; a 12 mm spot was used at a repetition rate of approximately 1 Hz, with contact cooling of the skin surface applied before each pulse to protect the epidermis.

All treatments were performed at room temperature by a single operator using fixed device settings within each group. After treatment, the HA material was recovered from the tissue for analysis. The HA depot was retrieved by incising the overlying skin with a scalpel and removing the gel bolus by sharp dissection; adherent tissue was gently detached and the recovered material was rinsed with phosphate-buffered saline to remove tissue debris before being subjected to the analytical assays.

### 2.5. Analytical Methods

#### 2.5.1. Fourier-Transform Infrared (FT-IR) Spectroscopy

**Figure 1:**
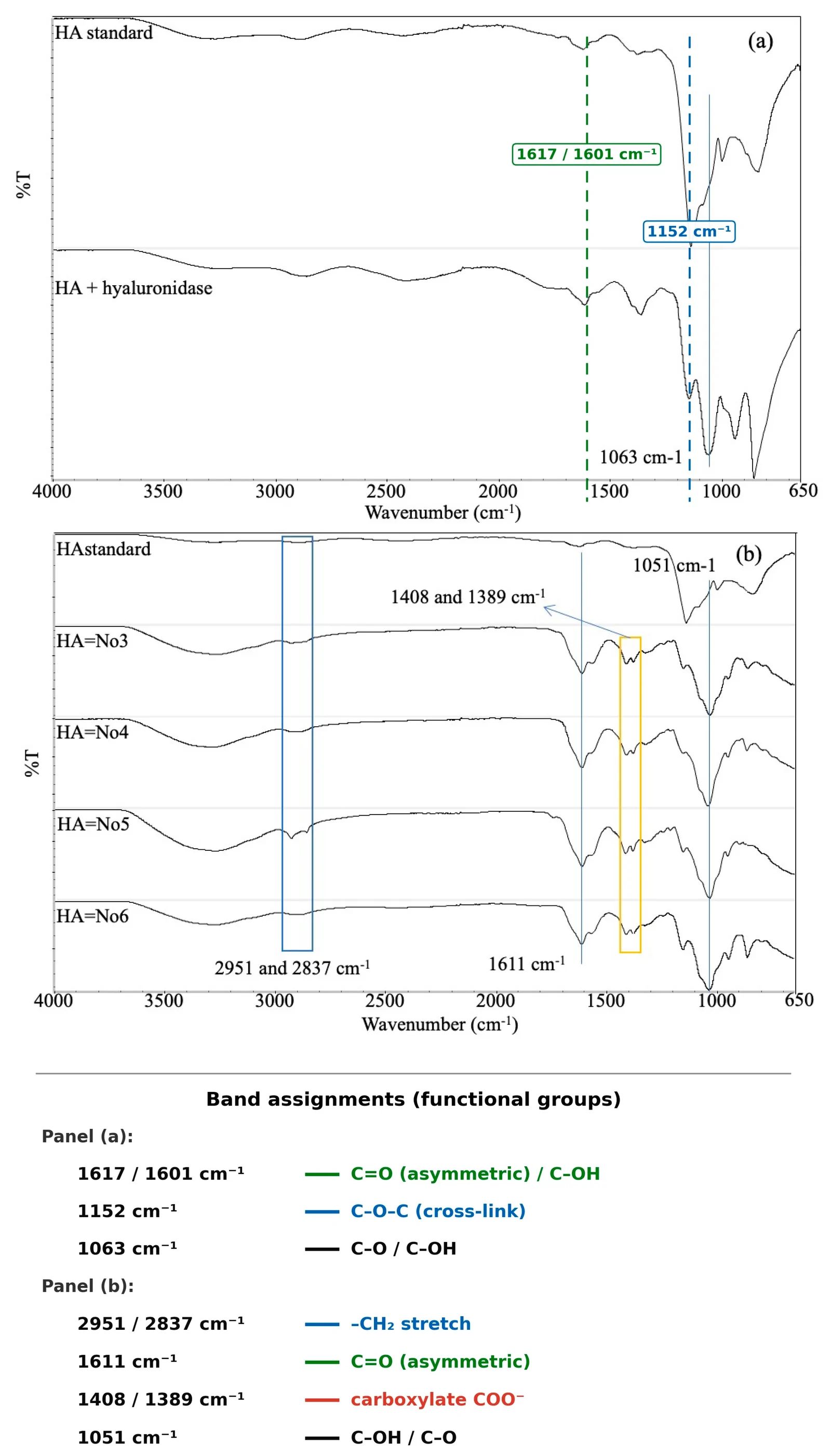
FT-IR spectra of HA-standard with (**a**) enzyme treatment, and (**b**) laser and sonication treatments. The principal absorption bands are annotated with their wavenumbers and functional-group assignments; in particular, the bands discussed in the text—including the C–O–C cross-link vibration near 1152 cm^−1^ and the C=O/C–OH region at 1617/1601 cm^−1^—are now indicated on panel (**a**).

The chemical structure of HA-idea before and after enzymatic, laser, and ultrasound treatment was characterised by FT-IR spectroscopy. Spectra were recorded over the 650–4000 cm^−1^ range at a resolution of 4 cm^−1^ using an FT-IR spectrometer fitted with an attenuated total reflectance (ATR) accessory (Thermo, Waltham, MA, USA, Nicolet iS10). Changes in the characteristic absorption bands of HA—including the aliphatic –CH_2_ stretching (2951–2837 cm^−1^), the asymmetric C=O vibration (≈1611–1617 cm^−1^), the C–OH stretching (≈1601 cm^−1^), the COO^−^ stretching (≈1409 cm^−1^), and the C–O–C band associated with cross-linking (≈1152 cm^−1^)—were used to assess treatment-induced structural modification. The band positions and their assignment to specific functional groups (the aliphatic –CH_2_ stretching, the amide/asymmetric C=O vibration, the symmetric COO^−^ stretching, the C–OH stretching and the C–O–C vibration associated with cross-linking) follow previously published infrared data for hyaluronan and its cross-linked derivatives [15,16,17,18]; these assignments are indicated directly on the spectra in Figure 1.

#### 2.5.2. Thermogravimetric Analysis (TGA)

The thermal stability of HA-idea treated with enzyme, laser, and ultrasound was investigated using a thermogravimetric analyser (TGA, SII TG/DTA 6300, Exstar, Seiko Instruments, Chiba, Japan). Measurements were carried out under a nitrogen atmosphere at a flow rate of 200 mL/min, with a heating rate of 10 °C/min from ambient temperature to 1000 °C. The onset temperatures and percentage weight losses of the successive degradation steps were determined from the resulting thermograms and used to compare the relative thermal stability of the samples.

#### 2.5.3. Hydrolytic Degradation of Irradiated and Sonicated Samples by HPLC

The hydrolytic degradation of the treated samples was investigated at pH 7.4 and 37.5 °C. Briefly, 0.5 mL of PBS was added to each irradiated/sonicated HA solution; 0.5 mL of the resulting solution was then withdrawn, further diluted with 2 mL of PBS, and transferred into a dialysis membrane. The membrane was placed into 25 mL of PBS in a shaking water bath maintained at 37.5 °C. The linear HA released by degradation was quantified by high-performance liquid chromatography (HPLC, Thermo Ultimate 3000) equipped with a refractive-index (RI) detector over a 3-day degradation period. Separation was performed on a Rezex RMM-Carbohydrate Na^+^ column (5 µm particle size, 300 mm × 7.8 mm i.d.5; Phenomenex). All analyses were conducted under identical conditions: a mobile phase of 5 mM sulfuric acid at a flow rate of 0.8 mL/min, a column temperature of 65 °C, an RI-detector temperature of 45 °C, an injection volume of 20 µL, and a run time of 10 min. The amount of degraded HA was calculated against a previously prepared HA calibration curve obtained under the same chromatographic conditions.

#### 2.5.4. Enzymatic Degradation by HPLC

The effect of hyaluronidase on the degradation of cross-linked HA-idea was evaluated and compared with the untreated control at pH 7.4 and 37.5 °C. Hyaluronidase (0.035 g) was dissolved in 2 mL of PBS; 1 mL of HA-idea was mixed with 2 mL of this enzyme solution and placed into a dialysis membrane, while the control consisted of 1 mL of HA-idea suspended in 2 mL of PBS. Each dialysis membrane was immersed in 25 mL of PBS in a shaking water bath at 37.5 °C, and the linear HA released over a 3-day period was quantified by HPLC using the chromatographic conditions described above. All treatments and subsequent assays were carried out in biological triplicate (*n* = 3). The HPLC degradation values are reported as mean ± standard deviation, whereas the FT-IR spectra and TGA thermograms shown are representative traces of the three replicate samples.

## 3. Results

### 3.1. FT-IR Spectroscopy

The chemical structure of the cross-linked hyaluronic acid before and after treatment was first examined by FT-IR spectroscopy, which probes the vibrational signatures of the functional groups most sensitive to cross-linking and chain scission. The FT-IR spectra of Sample 1 (untreated HA-idea) and Sample 2 (hyaluronidase-treated HA-idea) are given in Figure 1a. The distinct peak at 1152 cm^−1^ is attributed to the C–O–C band arising from the opening of the epoxy ring during cross-linking and its bonding with the –OH groups of HA; its presence therefore serves as a marker of the cross-linked network. The peaks observed at 1617 cm^−1^ in both samples are assigned to the asymmetric C=O vibration, and the band at 1601 cm^−1^ to the stretching of the C–OH group. The persistence of these characteristic bands in the enzyme-treated Sample 2 indicates that the fundamental saccharide backbone of HA is retained after exposure to hyaluronidase, consistent with a degradation that proceeds through hydrolysis of the glycosidic linkages rather than through destruction of the monosaccharide units.

**Scheme 1:**
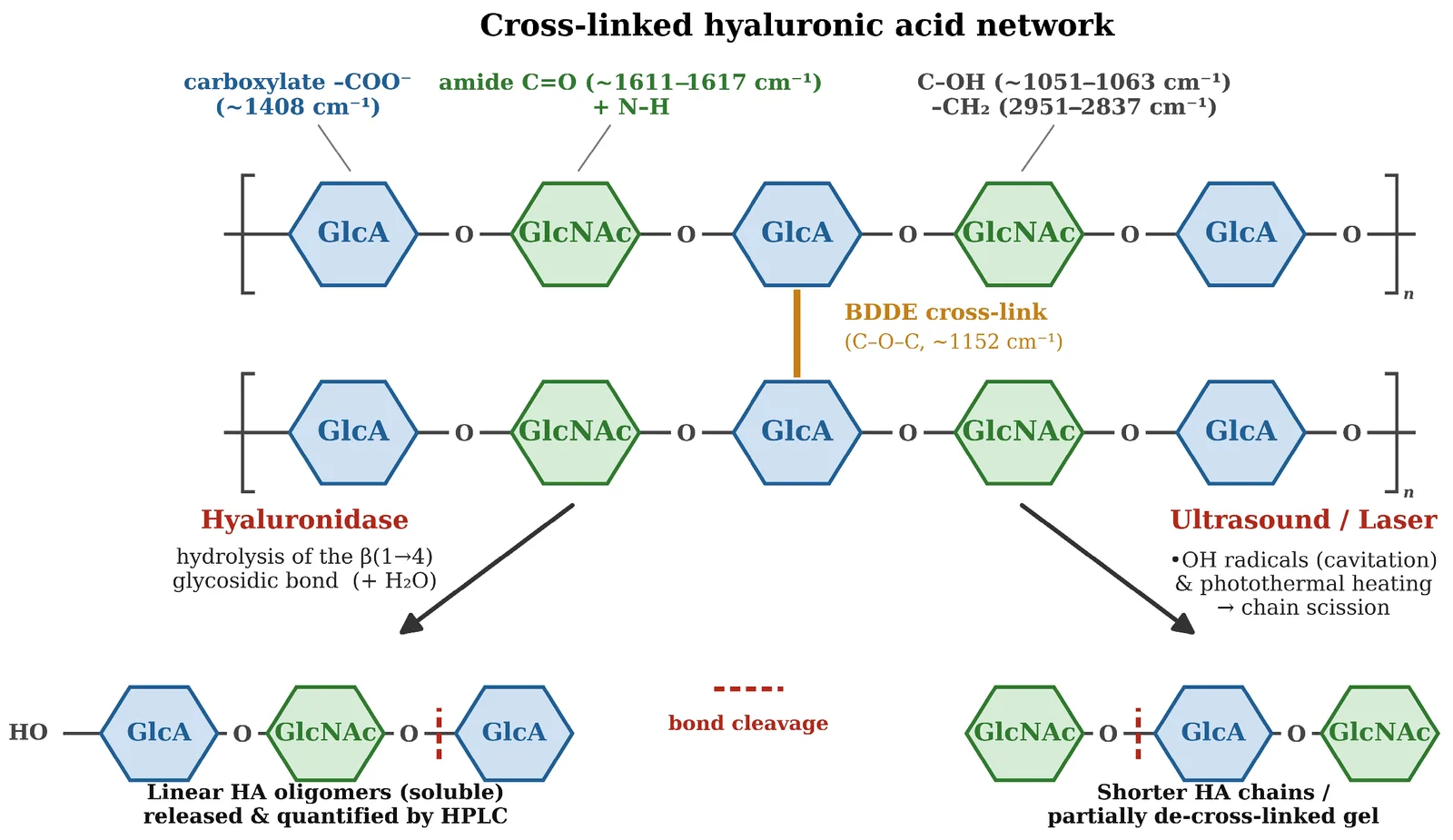
Proposed degradation pathways of the cross-linked hyaluronic acid network and their relationship to the FT-IR spectra. Hyaluronidase hydrolyses the β (1–4) glycosidic bond, whereas ultrasound (through hydroxyl radicals generated by acoustic cavitation) and laser irradiation (through photothermal heating) cleave the glycosidic linkages of the network. In every case the monosaccharide units are preserved, so the characteristic sugar-unit bands (amide C=O, carboxylate COO^−^, C–OH and –CH_2_) are retained in the infrared spectra, while the C–O–C cross-link band near 1152 cm^−1^ is attenuated, reflecting loss of cross-linking and shortening of the chains.

The FT-IR spectra of Sample 1 together with the ultrasound- and laser-treated samples (Samples 3–6) were then compared and are presented in Figure 1b. The spectrum of the untreated HA-idea is somewhat changed after laser and sonication treatment, and interpretable FT-IR spectra were obtained for all samples. In brief, the peaks observed at 2951–2837 cm^−1^ are attributed to the aliphatic –CH_2_ stretching, the band at 1611 cm^−1^ to asymmetric C=O vibrations, the band at 1409 cm^−1^ to the stretching of the COO^−^ group, and the band at 1051 cm^−1^ to C–OH stretching. The retention of these principal absorption bands across Samples 3–6 indicates that neither laser irradiation nor sonication abolishes the basic carboxylate and hydroxyl functionalities of HA; rather, the observed changes in relative band intensity and position are consistent with partial modification of the polymer network—most plausibly a reduction in the degree of cross-linking and a shortening of the chains—while the essential chemical identity of the hyaluronan is preserved. FT-IR therefore establishes that the treatments act by structural degradation of the polymer rather than by wholesale chemical transformation, and the extent of that degradation is quantified below by TGA and HPLC. Overall, these band positions and assignments agree with the infrared spectra reported for hyaluronan and for cross-linked HA fillers in the literature [15,16,17,18]. The attenuation and broadening of the C–O–C cross-link band near 1152 cm^−1^ is consistent with a partial loss of cross-linking; however, because ATR–FT-IR is not quantitative for cross-link density, this is interpreted only as qualitative evidence and is weighed together with the TGA and HPLC data rather than as a stand-alone measure of de-cross-linking. The chemistry that links glycosidic-bond cleavage to the retention of the backbone bands is summarised in Scheme 1.

### 3.2. Thermogravimetric Analysis

**Figure 2:**
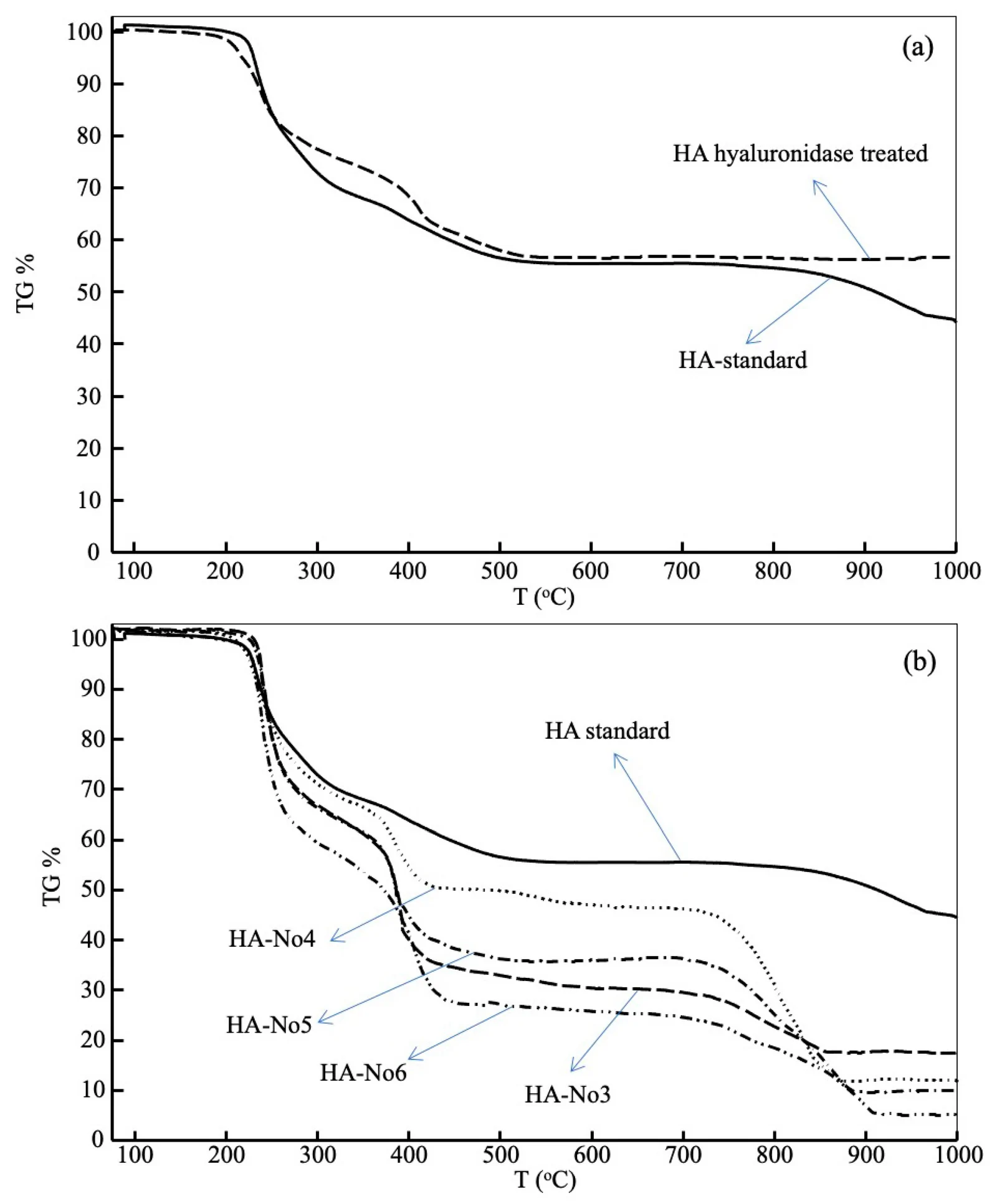
TGA thermograms of HA-standard with (**a**) enzyme treatment, and with (**b**) laser and sonication treatment.

The thermal stability of the untreated, enzyme-, ultrasound-, and laser-treated hyaluronic acid was compared, and the corresponding thermograms are presented in Figure 2; the derived onset temperatures and weight losses are summarised in Table 1. The thermal profile provides an indirect but sensitive measure of network integrity, since a more highly cross-linked, higher-molecular-weight polymer generally resists decomposition to higher temperatures.

As shown in Figure 2a, the first degradation step of untreated HA (Sample 1) began between 214 and 528 °C with a 43.2% weight loss, the second between 869 and 965 °C with a 54.3% weight loss, and at 1000 °C a total weight loss of 55.2% was obtained. The degradation of the hyaluronidase-treated HA (Sample 2) started at 193 °C and continued up to 263 °C with an 18.3% weight loss; a further step between 384 and 522 °C accounted for a 43.0% weight loss, and at 1000 °C a total weight loss of 43.4% was recorded. The appearance of an additional low-temperature decomposition step and the reduced total weight loss of Sample 2 are consistent with the generation of shorter, lower-molecular-weight hydrolysis fragments, together with a higher proportion of thermally stable residue remaining at 1000 °C.

The thermograms of the ultrasound- and laser-treated samples (Samples 3–6) are given in Figure 2b. The ultrasound-treated Sample 3 showed four degradation temperature ranges: 220–275 °C with a 29.3 wt% loss, 370–421 °C with a 64.0 wt% loss, 735–844 °C with an 81.2 wt% loss, and a final 82.5% weight loss at 1000 °C. The Nd:YAG-laser-treated Sample 4 likewise displayed four degradation steps: 220–275 °C with a 26.4 wt% loss, 366–417 °C with a 48.6 wt% loss, 725–887 °C with an 88.0 wt% loss, and 88.1% weight loss at 1000 °C. The diode-laser-treated Sample 5 began to degrade between 226 and 276 °C with a 30.0 wt% loss and continued at 362–425 °C with a 59.8 wt% loss and at 727–887 °C with a 90.1 wt% loss, remaining almost unchanged up to 1000 °C. Finally, the alexandrite-laser-treated Sample 6 showed a thermal-decomposition pattern closely resembling that of the other physically treated samples, beginning between 212 and 269 °C with a 35.5% weight loss and continuing through successive steps at 303–444 °C (72.2%), at 737–903 °C (93.7%), and reaching 94.9% weight loss at 1000 °C.

Taken together, the thermograms show that all of the physically treated samples underwent multi-stage decomposition with progressively greater total weight loss than the untreated and enzyme-treated materials, indicating a lower thermal stability that reflects treatment-induced disruption of the cross-linked network. The lowest thermal stability at 1000 °C was observed for the alexandrite-treated Sample 6, which exhibited the greatest cumulative weight loss (94.9%), whereas the highest thermal stability at 1000 °C was observed for the hyaluronidase-treated Sample 2, which retained the largest thermally stable residue (43.4% weight loss). This ordering suggests that the physical modalities and the enzymatic treatment degrade the polymer through distinct mechanisms: the former predominantly reduce network cross-linking and thermal resistance, whereas the latter cleaves the chains into fragments that leave a comparatively larger stable residue on pyrolysis. All TGA results are summarised in Table 1. The multi-step decomposition observed here—an initial low-temperature loss related to bound water and labile side groups, followed by the main pyrolytic breakdown of the polysaccharide backbone—is in good agreement with the thermogravimetric behaviour reported previously for hyaluronic acid and for its thermally and chemically degraded forms, in which chain scission and reduced molecular weight shift the decomposition steps and lower the residual mass [11,17].

### 3.3. Degradation Quantified by HPLC

**Figure 3:**
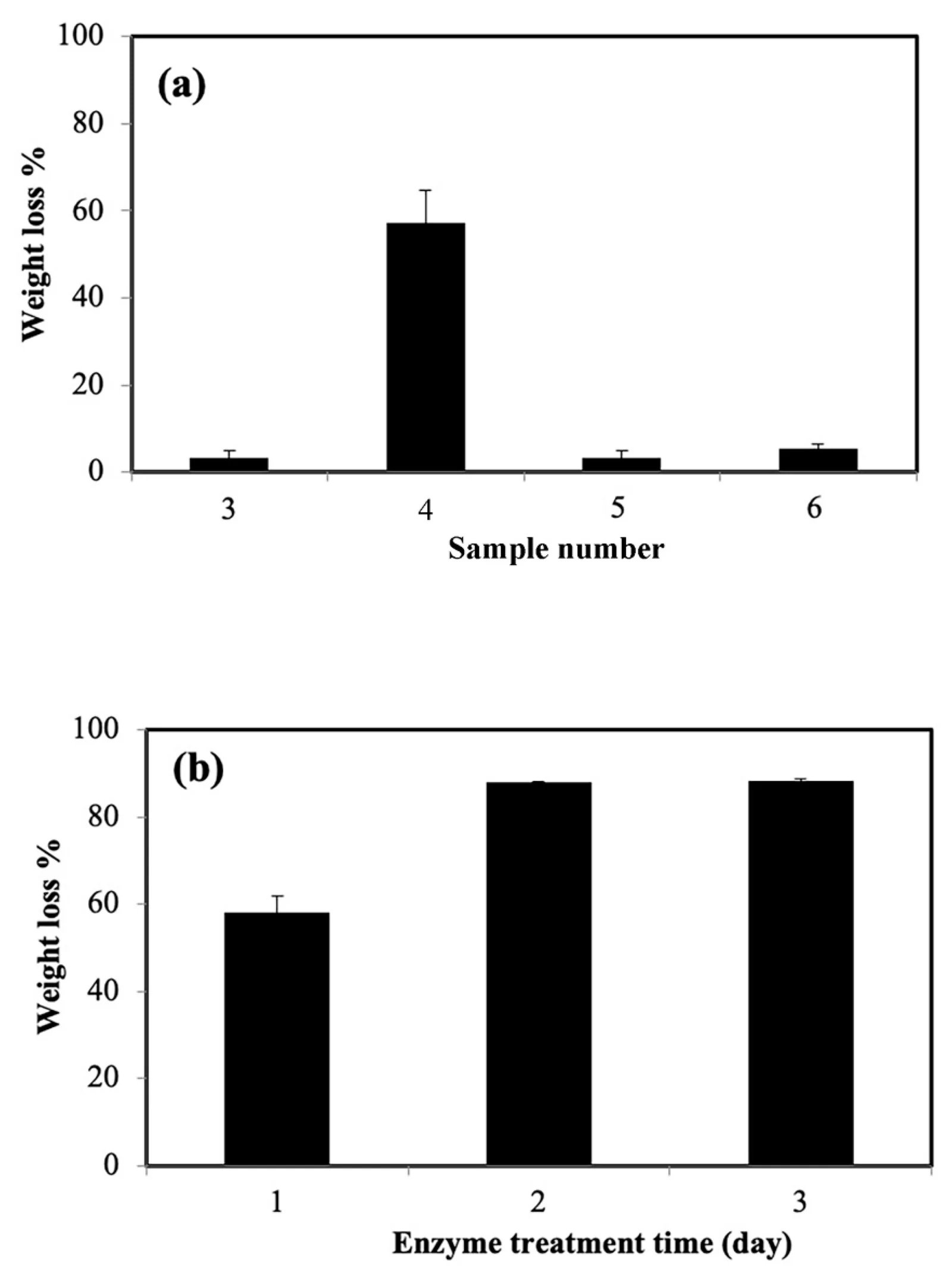
Degradation of cross-linked HA-idea treated with (**a**) different irradiation sources or time, and (**b**) hyaluronidase enzyme at 1-, 2- and 3-day treatments (degrading amounts were determined by HPLC).

The effects of ultrasound, laser irradiation, and enzymatic treatment on the degradation of cross-linked HA were further quantified by HPLC, which measures the proportion of the polymer released as soluble, linear HA and therefore provides a direct index of effective degradation. The weight-loss percentages of the ultrasound- and laser-treated samples are shown in Figure 3a. The ultrasound-treated Sample 3, the diode-laser-treated Sample 5, and the alexandrite-laser-treated Sample 6 did not show much degradation, with weight losses of 3.2 ± 1.1%, 3.2 ± 1.2%, and 5.3 ± 2.0%, respectively. In marked contrast, the Nd:YAG-laser-treated Sample 4 showed substantially greater degradation, with a 57.3 ± 5.2% weight loss. This pronounced difference indicates that, among the physical modalities examined, only the Nd:YAG laser (Sample 4) produced degradation of a magnitude comparable to enzymatic dissolution, whereas the ultrasound and the diode and alexandrite lasers caused only minor release of linear HA under the conditions tested—a finding consistent with the FT-IR evidence of only partial structural modification for those samples. It is also noteworthy that the alexandrite-treated Sample 6, despite showing the lowest thermal stability by TGA, released comparatively little soluble HA in the hydrolytic assay; this apparent dissociation reflects the fact that TGA and HPLC probe different properties—respectively, the thermal resistance of the residual network and the amount of chain material actually solubilised—and underscores the value of assessing degradation by complementary methods. It should be emphasised that the HPLC assay quantifies the fraction of HA released as soluble, low-molecular-weight material and therefore provides indirect evidence of chain scission rather than a direct determination of molecular weight; representative refractive-index chromatograms are provided in the Figure 1. A direct measurement of the native and degraded molecular-weight distribution—for example by size-exclusion or gel-permeation chromatography (SEC/GPC)—was beyond the scope of the present study and is acknowledged as a limitation (Section 4), so the conclusions regarding chain scission are drawn conservatively.

The enzymatic degradation of cross-linked HA by hyaluronidase (Sample 2) was evaluated after 1, 2, and 3 days of treatment, and the results are illustrated in Figure 3b. Cross-linked HA was degraded by 58.2 ± 2.9% at day 1, and the degradation increased to 87.8 ± 0.5% and 88.1 ± 0.9% at days 2 and 3, respectively. The enzymatic reaction thus reached the greater part of its ultimate effect within the first 24 h and approached a plateau by day 2, with little additional degradation between days 2 and 3. Notably, the single-day enzymatic weight loss (58.2 ± 2.9%) is of the same order as that produced by the most effective physical treatment, the Nd:YAG laser (Sample 4, 57.3 ± 5.2%), whereas the plateau value attained by hyaluronidase (≈88%) exceeded that of any single physical modality. This confirms that enzymatic degradation remained the most complete method overall, while identifying the Nd:YAG laser as the energy-based condition capable of a comparable initial effect and thus the most promising non-enzymatic alternative among those tested.

## 4. Discussion

The central observation of this study is easy to state but, we believe, not trivial: of the four energy-based conditions tested against a cross-linked hyaluronic acid filler, only the long-pulsed 1064 nm Nd:YAG laser degraded the polymer to a degree that approached enzymatic dissolution. The Nd:YAG-treated material released 57.3 ± 5.2% of its mass as soluble, linear HA in the hydrolytic HPLC assay, a value essentially indistinguishable from the 58.2 ± 2.9% achieved by hyaluronidase after 24 h. In contrast, microfocused ultrasound (3.2 ± 1.1%), the diode laser (3.2 ± 1.2%), and the alexandrite laser (5.3 ± 2.0%) left the great majority of the gel intact. Hyaluronidase nonetheless remained the most complete method overall, reaching a plateau of roughly 88% by the second day. The practical reading is therefore twofold: the enzyme retains its place as the reference standard, but a specific, widely available laser wavelength is capable of producing a first-day effect of the same order—and that is worth taking seriously in a field that currently has essentially one tool for dissolving filler.

Why the 1064 nm wavelength should succeed where 755 nm and the diode laser fail is, we think, best explained by where each wavelength deposits its energy. Cross-linked HA fillers are hydrogels that are approximately 90% water, so for a purely photothermal effect the relevant chromophore inside the depot is water rather than melanin or haemoglobin [11]. Across the near-infrared, water absorption is not flat: it rises markedly beyond about 950 nm, so that 1064 nm light is absorbed by water roughly an order of magnitude more strongly than light at 755–808 nm [19]. At the same time, 1064 nm is scattered less and is poorly absorbed by epidermal melanin, which allows it to reach a target seated 2–3 mm deep at the dermal–subcutaneous junction—precisely the plane into which the filler was injected. The alexandrite (755 nm) and diode lasers, by contrast, are engineered around melanin absorption for hair removal; their energy is preferentially taken up superficially by pigment and comparatively little is absorbed by the water of a deep gel bolus. In the framework of selective photothermolysis, all three lasers were “on target” for their usual clinical chromophore, but only the Nd:YAG was on target for water [20].

Add to this the 150 ms pulse used here—long enough to act as bulk heating rather than a brief microsecond pulse—and the Nd:YAG condition becomes the one most likely to raise the temperature of the whole depot past the 56–90 °C window in which cross-linked HA thermally destabilises [11]. The result is consistent with, and arguably predictable from, the physics; what the experiment adds is a direct quantification of how large the difference between wavelengths actually is.

A more subtle point concerns the apparent disagreement between our two structural read-outs. By thermogravimetry the alexandrite-treated sample was the least thermally stable of all, losing 94.9% of its mass by 1000 °C, yet by HPLC it released almost no soluble HA. This dissociation is a useful reminder that TGA and HPLC do not measure the same thing. Thermogravimetry reports how the recovered solid decomposes on pyrolysis, and is sensitive to changes in the network—surface charring, altered cross-link density, or a shifted water-binding profile—that need not correspond to any chain material actually being liberated into solution.

HPLC, on the other hand, counts only what has been depolymerised sufficiently to diffuse through a dialysis membrane, which is much closer to the clinically meaningful endpoint of “filler that has been removed.” The FT-IR data sit comfortably with this interpretation: across all the physically treated samples the characteristic carboxylate, hydroxyl and C–O–C cross-linking bands were retained, with only modest changes in relative intensity, indicating partial modification of the network rather than wholesale scission. Taken together, the three techniques argue that superficial or thermal alteration of a gel is not the same as effective degradation, and that thermal stability alone would have been a misleading surrogate had we relied on it in isolation.

The failure of microfocused ultrasound to degrade the gel deserves comment, because the sonochemical literature might have predicted otherwise. Hyaluronic acid is genuinely susceptible to ultrasonic depolymerisation, but the efficient degradation described in physicochemical studies typically depends on high-molecular-weight substrate in dilute solution, prolonged exposure, and often the deliberate addition of hydrogen peroxide or transition-metal ions to amplify radical production; even then, the mechanical, cavitation-driven pathway dominates for large polymers while radical and thermal pathways matter more for smaller chains [10]. A clinical microfocused device operating at 7 MHz delivers brief, tightly focused thermal pulses over a few milliseconds, in a densely cross-linked gel where cavitation behaves very differently from a stirred aqueous solution. Under those conditions it is not surprising that little soluble HA was released. This does not mean HIFU cannot affect filler—the very fact that thermal interaction between energy devices and HA has been flagged as a clinical concern shows that it can under some settings [11,12]—but it does suggest that the energy, dwell time and coupling used in a standard tightening protocol are far from optimised for degradation. Our negative result is therefore best read as parameter-dependent rather than as a statement about ultrasound in principle.

These findings also help to reconcile a tension in the existing laser literature. Earlier work, most notably the porcine model of Alam and colleagues, was designed to reassure clinicians that common laser and light treatments do not damage nearby filler, and reported minimal interaction at typical clinical settings [13]. That is not in conflict with what we observed. The same 1064 nm wavelength that is “safe” beside filler at conservative parameters can degrade it once the delivered thermal dose is high enough—a difference in settings, not of physics. The isolated reports of intralesional 1444 nm Nd:YAG used to reduce filler granulomas point in the same direction, treating the laser as a tool to shrink an unwanted deposit rather than as a bystander [14]. Our data give that intuition a quantitative, comparative footing and identify the 1064 nm long-pulsed Nd:YAG, specifically, as the most promising non-enzymatic candidate among the modalities we compared.

Clinically, the appeal of a laser-based alternative is not that it could replace hyaluronidase outright—it plainly cannot, at least not on these numbers. The enzyme was both more complete and, in the acute setting where it matters most, far faster; nothing here challenges its role in the emergency management of vascular occlusion, where speed and diffusibility are decisive [4]. The interest lies at the margins the enzyme handles poorly. A patient with documented hypersensitivity to hyaluronidase, including those sensitised to hymenoptera venom in whom the enzyme is an allergen, currently has few good options [8]; a physical modality that does not introduce a foreign protein is attractive precisely there. So too is the prospect of a spatially controlled reduction of a discrete, superficial nodule without the diffuse, non-selective loss of native HA that an over-generous dose of enzyme can cause [5,6]. But the same mechanism that makes 1064 nm effective is also its principal hazard: bulk heating of a water-rich target 2–3 mm deep is not selective for the filler, and in living tissue that heat would be shared with the surrounding dermis and subcutis, with a real risk of thermal injury, scarring and dyspigmentation. Any translation to patients will stand or fall on whether a therapeutic window exists between degrading the gel and burning the tissue around it. The two classes of method also differ in the nature of their selectivity. Hyaluronidase has essentially no molecular selectivity, since it degrades native and injected HA alike; a focused energy source offers spatial selectivity, in that it can be aimed at a defined deposit, but likewise no molecular selectivity, because any HA and any water-rich tissue within the treated volume are heated together. Neither modality is therefore intrinsically selective for the filler, and this contrast between molecular and spatial selectivity is central to how a non-enzymatic method might be positioned clinically.

Several limitations bound these conclusions. The study is ex vivo: porcine ear skin is a well-validated surrogate for human skin in structure and in penetration studies, and it allowed the filler to be placed at a realistic depth [21], but it lacks perfusion, lymphatic clearance and any inflammatory or enzymatic response, all of which shape how filler is actually eliminated in vivo. We examined a single commercial filler with one cross-linking chemistry; because resistance to degradation scales with cross-link density and formulation, the ranking we observed may shift for more or less heavily cross-linked products [22]. Degradation was quantified with surrogate physicochemical assays rather than clinical or histological endpoints, and we did not record intra-tissue temperatures, so the thermal argument, though well supported, remains inferential. An important limitation is that none of the three techniques applied here measures molecular weight directly: FT-IR, TGA and HPLC provide complementary but indirect evidence of degradation, so chain scission is inferred rather than demonstrated. A direct determination of the native and degraded molecular-weight distribution by size-exclusion or gel-permeation chromatography (SEC/GPC), and a complementary differential scanning calorimetry (DSC) analysis of the thermal transitions, were beyond the scope of this ex vivo study; both would be required to confirm the extent of chain scission, and the conclusions have accordingly been kept moderate. The make, origin and activity of the hyaluronidase preparation are specified in Section 2. Finally, replicate numbers were modest and treatments were performed by a single operator, so the absolute percentages should be read as indicative rather than definitive.

The obvious next steps follow directly. A dose–response study for the 1064 nm Nd:YAG—varying fluence, pulse duration and pulse number while measuring both HA degradation and in situ temperature—would define whether a usable therapeutic window exists, and repeating the comparison across fillers of differing cross-link density would test how general the wavelength effect is [22]. In vivo work with histological safety endpoints is essential before any clinical claim, and a combination approach, using sub-ablative laser heating to soften a resistant, highly cross-linked bolus so that a smaller dose of hyaluronidase can finish the job, may prove more practical than either method alone. For now, the honest summary is that hyaluronidase remains the standard, that ultrasound and the melanin-targeting lasers offered little at the settings tested, and that the 1064 nm Nd:YAG laser is the one non-enzymatic modality from this comparison worth pursuing further.

## 5. Conclusions

Within the limits of this ex vivo model, enzymatic degradation with hyaluronidase remained the most effective and most complete method of dissolving cross-linked hyaluronic acid, reaching approximately 88% degradation by the second day and confirming its position as the reference standard. Among the non-enzymatic, energy-based modalities, however, the long-pulsed 1064 nm Nd:YAG laser stood apart: it degraded the filler by 57.3 ± 5.2%, a first-day effect comparable to that of hyaluronidase (58.2 ± 2.9%), whereas microfocused ultrasound (Ulthera) and the alexandrite and diode lasers produced only marginal release of soluble HA (3.2–5.3%) under the settings tested.

The wavelength dependence of this effect is consistent with a photothermal mechanism in which water, the dominant constituent of the hydrogel, is the operative chromophore: the strong water absorption and favourable depth of penetration of 1064 nm light, combined with a long 150 ms pulse, allow bulk heating of the deep filler depot above its thermal-destabilisation threshold, while the melanin-targeting alexandrite and diode wavelengths deposit little energy in the water-rich gel. The dissociation observed between thermogravimetric and chromatographic results further indicates that thermal or superficial modification of the gel should not be equated with clinically meaningful degradation, and that effective removal is best judged by the amount of HA actually solubilised.

Taken together, these findings identify the 1064 nm Nd:YAG laser as the most promising non-enzymatic alternative for degrading hyaluronic acid filler and a rational candidate for further study—particularly for patients in whom hyaluronidase is contraindicated, such as those with hypersensitivity to the enzyme, or where a spatially controlled reduction of a discrete deposit is desired. It does not, on present evidence, replace hyaluronidase, which remains faster and more complete and irreplaceable in the emergency management of vascular complications. Because the same bulk heating that degrades the gel is not selective for it, the principal question for translation is whether a therapeutic window exists between degrading the filler and injuring the surrounding tissue. Controlled dose–response studies of the Nd:YAG laser with in situ thermometry, evaluation across fillers of differing cross-link density, and in vivo work with histological safety endpoints are the necessary next steps, and a combined strategy—sub-ablative laser heating followed by a reduced dose of hyaluronidase—merits particular attention. Finally, these conclusions are necessarily restricted to the single cross-linked filler, the specific device settings and the ex vivo model studied here, and should not be generalised to all hyaluronic acid fillers; because resistance to degradation depends on the cross-linking chemistry and cross-link density, the ranking observed may differ for other products, and confirmation across a range of fillers and under in vivo conditions is required before any clinical recommendation can be made.

## Figures and Tables

**Table 1:** Degradation temperature intervals with degradation amount of HA-idea with enzyme, laser irradiation and sonication.

Material	Thermal Degradation Steps (°C) and Weight Loss (%)			
	I	II	III	IV
HA-standard (control)	214–528 43.2%	868–965 54.3%	1000 55.2%	
HA-enzyme	193–263 18.3%	384–522 43.0%	1000 43.4%	
HA-No.3 microfocused ultrasound	220–275 29.3%	370–421 64.0%	735–844 81.2%	1000 82.5%
HA-No.4 Nd:YAG 1064 nm	196–283 26.4%	366–417 48.6%	725–871 88.0%	1000 88.1%
HA-No.5 diode laser 808 nm	226–276 30.0%	362–425 59.8%	727–887 90.1%	1000 90.1%
HA-No.6 alexandrite laser 755 nm	212–269 35.5%	303–444 72.2%	737–903 93.7%	1000 94.9%

## Data Availability

The data presented in this study are available on request from the corresponding author.
